# From Allozymes to Genomics: Reframing the Systematics and Population Structure of *Opisthorchis viverrini* and Its *Bithynia* Hosts

**DOI:** 10.3390/biology15131018

**Published:** 2026-06-26

**Authors:** Naruemon Bunchom, Weerachai Saijuntha, Paiboon Sithithaworn, Ross H. Andrews, Alan D. Ziegler, Chairat Tantrawatpan

**Affiliations:** 1 Center of Excellence in Biodiversity Research, Mahasarakham University, Maha Sarakham 44150, Thailand; aoy_narumon@hotmail.com; 2 Faculty of Medicine, Mahasarakham University, Maha Sarakham 44000, Thailand; 3 Cholangiocarcinoma Research Institute, Khon Kaen University, Khon Kaen 40002, Thailand; paib_sit@kku.ac.th (P.S.); rhandrews@gmail.com (R.H.A.); 4 Department of Parasitology, Faculty of Medicine, Khon Kaen University, Khon Kaen 40002, Thailand; 5 Department of Surgery & Cancer, Faculty of Medicine, Imperial College, London SW7 2AZ, UK; 6 Andaman Coastal Research Station for Development, Faculty of Fisheries, Kasetsart University, Ranong 85120, Thailand; thaihawk@gmail.com; 7 Division of Cell Biology, Department of Preclinical Sciences, Faculty of Medicine, and Center of Excellence in Stem Cell Research and Innovation, Thammasat University, Rangsit Campus, Pathum Thani 12120, Thailand

**Keywords:** cholangiocarcinoma, co-evolution, genetic structure, genetic variation, hydrological system, intermediate host, Lower Mekong Basin, opisthorchiasis

## Abstract

The carcinogenic liver fluke *Opisthorchis viverrini* underlies one of the world’s heaviest burdens of bile duct cancer, yet for decades it was treated as a single, genetically uniform parasite whose transmission was shaped mainly by environment and human behavior. However, advances in molecular biology have fundamentally reshaped this conceptual model. Evidence accumulated over the past three decades demonstrates that *O. viverrini* comprises geographically structured populations linked to hydrological connectivity, host distribution, and long-term evolutionary processes across interconnected river systems of mainland Southeast Asia, particularly within the Lower Mekong Basin. This review synthesizes research on the systematics and population structure of *O. viverrini* and its *Bithynia* snail hosts, tracing the transition from early allozyme studies to contemporary DNA-based and genomic approaches. Collectively, mitochondrial, nuclear, microsatellite, and intron markers reveal strong spatial structuring among parasite populations, while genetic patterns observed in snail hosts show partial geographic concordance with parasite population structure, suggesting that both may be influenced by shared hydrological organization, ecological isolation, and host connectivity across endemic aquatic systems. Population structure is strongly scale-dependent, with local panmixia often occurring within connected aquatic systems but pronounced differentiation emerging across broader geographic regions. Together, these findings indicate that transmission dynamics are shaped not only by environmental and behavioral factors, but also by evolutionary and landscape-level processes influencing host and parasite connectivity. Finally, we emphasize the increasing significance of population genomics and landscape genetics in understanding how transmission systems persist, disperse, reconnect, and respond to environmental change across endemic landscapes.

## Simple Summary

The liver fluke *Opisthorchis viverrini* is a parasitic worm that infects millions of people in Southeast Asia and is a major cause of bile duct cancer. For many years, scientists believed that this parasite was genetically similar throughout its distribution. However, recent studies using DNA and genomic technologies have shown that different populations exist across the Lower Mekong region. This review summarizes how research has advanced from early genetic methods to modern genomic approaches and what these studies have revealed about the parasite’s diversity. The findings show that parasite populations are influenced by geography, river systems, environmental conditions, and the distribution of their snail hosts. Understanding these genetic differences is important because they can affect how the parasite spreads, adapts to local environments, and persists in endemic areas. This knowledge will help improve future studies of parasite transmission, evolution, and disease control.

## 1. Introduction

The food-borne liver fluke *Opisthorchis viverrini* (Poirier, 1886) Stiles & Hassall, 1896 is among the most medically important species of trematode parasite in the Greater Mekong Subregion [1]. Chronic infection with *O. viverrini* is a major risk factor for cholangiocarcinoma (CCA), a highly aggressive hepatobiliary cancer [2]. Extensive epidemiological, clinical, and experimental studies have demonstrated a causal link between prolonged opisthorchiasis and malignant transformation of the bile duct epithelium. Consequently, *O. viverrini* has been classified as a Group 1 biological carcinogen by the International Agency for Research on Cancer [3], placing it among the few helminth parasites with confirmed carcinogenicity in humans [4].

Transmission of opisthorchiasis is sustained by a complex multi-host life cycle involving freshwater snails of the genus *Bithynia* (Leach, 1818) as first intermediate hosts, and cyprinid fish as second intermediate hosts, in which infective metacercariae develop [5]. Humans and other fish-eating mammals serve as definitive hosts, in which adult flukes inhabit the bile ducts, reproduce sexually, and shed eggs into the environment through feces [6]. Human infection occurs through the consumption of raw or undercooked freshwater fish containing metacercariae, a transmission route closely linked to culinary traditions deeply rooted in the region, where raw or undercooked fish dishes retain great cultural significance [7,8].

*Opisthorchis viverrini* is endemic across multiple major river systems of mainland Southeast Asia, including Thailand, Lao PDR, Cambodia, Vietnam, and fringe areas of Myanmar [9]. However, the principal historical endemic focus occurs within the Lower Mekong Basin, particularly northeastern Thailand and adjacent tributary systems in adjacent areas of Lao PDR, where long-standing floodplain connectivity, wetland systems, and fish-based transmission cycles have supported persistently high infection prevalence [10]. Beyond this core region, prevalence generally declines and becomes more spatially heterogeneous across peripheral basins in Cambodia and Vietnam, while evidence from frontier regions of Myanmar remains comparatively sparse and uncertain [9]. These gradients likely reflect interacting differences in geophysical boundaries, ecohydrological connectivity, intermediate-host distribution, and uneven historical surveillance effort. 

Despite decades of public-health interventions, opisthorchiasis remains a major health problem in endemic areas [6]. Control programs focusing on mass drug administration, sanitation improvements, and public education have reduced infection intensity in some areas but have not eliminated transmission [11]. In contemporary endemic settings, persistent transmission is maintained by humans and domestic animals consuming raw or undercooked fish and releasing parasite eggs into aquatic environments through inadequate sanitation practices [12,13]. This human-to-environment linkage effectively sustains the transmission cycle, which historically depended more heavily on wild fish-eating mammals [14].

For much of the twentieth century, *O. viverrini* was generally regarded as a single morphologically defined species distributed across mainland Southeast Asia [7,15]. Because taxonomic studies relied primarily on adult morphology, potential cryptic diversity and fine-scale population subdivision remained largely unresolved (e.g., Vajrasthira et al. [16]; Wykoff et al. [17]). Consequently, geographic variation in infection prevalence was interpreted primarily in relation to environmental, ecological, and behavioral factors rather than plausible genetic differentiation among parasite populations [6,18].

However, advances in molecular biology over the past two decades have altered this perspective. Increasing evidence from population genetic and phylogenetic studies indicates that *O. viverrini* exhibits substantial genetic structure across its geographic range. Rather than representing a single panmictic species, the parasite comprises multiple genetically differentiated lineages associated with major river systems [18]. Simultaneously, molecular studies of the snail intermediate hosts have revealed substantial genetic diversity and strong population subdivision. Snails traditionally classified as *B. siamensis* sensu lato are now recognized to represent a species complex comprising multiple divergent evolutionary lineages, many of which are associated with specific river catchments [19,20,21].

These discoveries highlight the importance of hydrological connectivity and river basin organization in shaping both host and parasite population structures over evolutionary timescales. The emerging picture is that the transmission of occurs within spatially structured host–parasite assemblages embedded within interconnected aquatic landscapes. Concordant geographic structuring between parasite and snail populations further suggests long-term coevolutionary relationships shaped by ecological isolation, drainage connectivity, and historical landscape processes [12,18].

In this review, we synthesize over two decades of research on the systematics and population genetics of *O. viverrini* and its *Bithynia* snail hosts. Relevant literature published between 2000 and 2026 was identified through searches of Web of Science, Scopus, PubMed, and Google Scholar using combinations of the keywords “*Opisthorchis viverrini*”, “*Bithynia*”, “population genetics”, “genetic variation”, “genetic diversity”, “phylogeography”, “microsatellite”, “mitochondrial DNA”, “nuclear markers”, “allozymes”, “genomics”, and “Lower Mekong Basin”. Additional articles were identified through citation tracking of relevant publications and review papers.

Studies were included if they reported original genetic data or provided significant methodological or conceptual advances related to the population structure, genetic diversity, phylogeography, host–parasite interactions, or genomic analyses of *O. viverrini* and/or *Bithynia* snails. Studies focusing exclusively on clinical, epidemiological, or diagnostic aspects without a population genetic component were excluded. The retrieved literature was evaluated and synthesized qualitatively, with emphasis on the development of molecular markers, major findings, methodological advances, and emerging research directions.

As a narrative review, this study does not aim to provide a systematic analysis. Instead it presents a comprehensive synthesis of the evolution of population genetic research in this host–parasite system over the past two decades. We trace the methodological transition from early allozyme studies to contemporary genomic approaches and discuss how these advances have reshaped our understanding of parasite diversity, host–parasite associations, and transmission ecology within the Lower Mekong Basin. Finally, we explore how emerging genomic and landscape genetic frameworks may provide new insights into host–parasite coevolution, transmission dynamics, and the ecological and evolutionary processes shaping opisthorchiasis across endemic landscapes.

## 2. Early Molecular Insights: Allozyme Studies

The first molecular evidence challenging the traditional view of *O. viverrini* as a genetically homogeneous species emerged from allozyme electrophoresis studies conducted in the late twentieth century [22]. That study used three enzyme markers to examine *O. viverrini* specimens obtained from a human autopsy, revealing detectable enzymatic variation among parasites and suggesting the potential for underlying genetic differentiation. However, the limited number of loci and uncertainty regarding the broader population-level utility of these markers constrained interpretation of the results. Prior to the widespread adoption of DNA sequencing technologies, such allozyme-based approaches provided one of the few practical methods available for assessing genetic variation in parasitic organisms. Although limited by modern standards, these early studies established an important foundation for identifying population-level genetic structure in *O. viverrini*.

The first population-level allozyme analyses of *O. viverrini* revealed substantial polymorphism across multiple enzyme loci among parasites collected from different regions of Thailand [23]. Significant differences in allele frequencies among geographic populations provided the first indication that the parasite was not genetically uniform but instead exhibited measurable population subdivision. These findings marked a conceptual shift away from the long-standing assumption of panmixia (i.e., a single genetically well-mixed population), suggesting that ecological or geographic barriers could influence patterns of parasite dispersal and gene flow. Importantly, these early studies demonstrated that even morphologically indistinguishable parasites could harbor underlying genetic differentiation, raising the possibility of cryptic population structure.

Subsequent investigations expanded both the geographic scope and the number of independent loci examined. In some cases, as many as 32 enzyme loci were examined across *O. viverrini* and *Bithynia* snail populations from northeastern Thailand and Lao PDR [23,24]. These studies consistently reported significant genetic differentiation among parasite populations, with patterns indicative of restricted gene flow across landscapes. In parallel, comparable genetic structuring was observed in *Bithynia* snails, suggesting that shared environmental and geographical constraints may influence both host and parasite populations.

One plausible explanation for these patterns is that hydrological connectivity influences the dispersal of parasite hosts differently across landscapes of varying scale. Because the life cycle of *O. viverrini* depends on aquatic hosts, parasite movement is closely linked to the dispersal capacities and habitat distributions of both snails and fish. River networks may facilitate connectivity within floodplains and connected wetlands while limiting movement between isolated catchments or steep upstream environments. In particular, the more limited dispersal capacity and habitat specificity of *Bithynia* snails may contribute disproportionately to long-term population subdivision and localized host–parasite associations over evolutionary timescales.

However, evidence from finer-scale studies revealed a more complex and scale-dependent pattern of population structure. A longitudinal study examining *O. viverrini* collected from four species of cyprinid fish in Khon Kaen Province over a four-year period reported very low fixation index (*F*_ST_) values, indicating little genetic differentiation among parasite populations across sampling years and host species [23,24,25]. These results indicate that within a single hydrologically connected reservoir system, parasite populations may experience high local gene flow and approach panmixia—in contrast to the first-intermediate host *Bithynia siamensis* sensu lato, which was later shown to have single-lineage occupancy at the same local scale.

Occasional heterozygote deficiencies observed in these studies were interpreted as potential signatures of self-fertilization [24,25], a reproductive strategy known in many trematodes [26]. Alternatively, these patterns may reflect ecological variation in host availability or transmission intensity. Together, these findings highlight that *O. viverrini* population structure is not uniform but varies across spatial scales, with local mixing occurring within connected habitats and genetic differentiation emerging across broader geographic regions.

Despite their important contributions, allozyme markers have inherent methodological limitations. Their relatively low resolution, dependence on a limited number of protein-coding loci, and inability to detect synonymous or non-coding variation restrict their capacity to capture the full extent of genetic diversity. In addition, factors such as enzyme instability, scoring ambiguity, and potential selection acting on coding loci may bias estimates of population structure. However, early allozyme-based studies provided the first evidence of geographically structured genetic differentiation in *O. viverrini* and its associated *Bithynia* hosts [18,24].

These limitations ultimately motivated the transition toward DNA-based molecular markers, which offer substantially higher resolution and have enabled more robust investigation of parasite population structure and phylogeography. Nevertheless, allozyme studies played a pivotal role in the development of molecular parasitology in *O. viverrini* by laying the conceptual and methodological foundation for subsequent DNA-based investigations of parasite population structure and transmission dynamics.

## 3. DNA-Based Molecular Markers

### 3.1. Mitochondrial and Nuclear Ribosomal DNA Markers

The introduction of polymerase chain reaction (PCR) and DNA sequencing technologies marked a major turning point in the study of genetic variation in *O. viverrini* and its intermediate hosts. Compared with allozyme markers, DNA-based approaches provided substantially higher resolution and enabled the direct examination of nucleotide variation across multiple genomic regions. This transition allowed researchers to move beyond detecting broad patterns of differentiation toward reconstructing phylogeographic structure, inferring evolutionary relationships, and identifying cryptic diversity within parasite populations.

Among the various molecular markers employed, mitochondrial DNA (mtDNA), particularly the cytochrome c oxidase subunit 1 (*CO1*) and NADH dehydrogenase subunit 1 (*ND1*) genes, has been widely used to investigate the population genetics of *O. viverrini*. Most mitochondrial genes have revealed relatively low levels of variation within *O. viverrini* populations [18]. Nevertheless, mtDNA has proven valuable for assessing genetic diversity, reconstructing phylogenetic relationships, and identifying broad-scale patterns of population structure. As a maternally inherited, non-recombining marker, however, mtDNA represents only a single genealogical history and may not fully capture the evolutionary processes shaping population differentiation. Furthermore, processes such as incomplete lineage sorting, introgression, and selective sweeps can generate discordance between mitochondrial and nuclear genetic patterns, potentially leading to incomplete interpretations of population history when mtDNA is used in isolation [27]. Consequently, although mitochondrial markers have provided important insights into the genetic diversity and population structure of *O. viverrini*, the integration of multiple independent loci can provide a more comprehensive understanding of evolutionary relationships and population processes [28,29].

Recent studies have further highlighted both the utility and limitations of mtDNA markers in *O. viverrini*. A multilocus analysis combining mitochondrial (*CO1* and *ND1*) and nuclear markers demonstrated clear genetic structuring within the *O. viverrini* species complex and identified a distinct lineage from Sakon Nakhon Province, Thailand, that may represent a cryptic species [30]. Notably, genetic differentiation in this study was detected primarily using mitochondrial markers, whereas the nuclear loci examined showed little or no variation. In contrast, a population genetic study based on mitochondrial *CO1* and *ND1* sequences from northern Thailand revealed low and non-significant genetic differentiation among populations, accompanied by high estimated gene flow and signatures of recent demographic expansion [31].

Collectively, these studies demonstrate that the extent of population structure detected by mtDNA markers can vary depending on the geographic scale, demographic history, and evolutionary context of the populations examined. Consequently, mitochondrial markers remain valuable tools for investigating genetic diversity and population structure in *O. viverrini*, but their interpretations should be made cautiously when used in isolation. This is particularly important for trematodes with complex life cycles, where multiple hosts and ecological interactions may influence patterns of gene flow and population connectivity [18,32]. Integrating mtDNA with nuclear and genomic markers can therefore provide a more comprehensive and robust understanding of population structure and evolutionary history. Despite these limitations, mitochondrial genes remain highly useful for molecular identification and species discrimination. In particular, they have proven effective for distinguishing *O. viverrini* from closely related opisthorchiid species and from morphologically similar *Opisthorchis*-like eggs of minute intestinal flukes, such as *Haplorchis taichui* [31,33]. 

To address these limitations, nuclear DNA markers have been increasingly incorporated into population genetic studies of *O. viverrini*. Ribosomal DNA regions, particularly the internal transcribed spacers (ITS1 and ITS2), have been used to complement mitochondrial data and provide additional resolution for species identification and population differentiation [34,35]. Although ITS markers generally exhibit lower variability than mtDNA, they have proven useful in confirming patterns of genetic subdivision and in distinguishing closely related taxa. Importantly, congruence between mitochondrial and nuclear markers has strengthened evidence for genuine population structuring rather than artefacts of marker-specific biases [36]. In particular, ITS2-based molecular approaches have demonstrated high diagnostic value in differentiating *O. viverrini* from morphologically similar minute intestinal flukes (MIFs), especially *H. taichui*, thereby reducing misclassification of *Opisthorchis*-like eggs in epidemiological studies [34,35]. However, evidence from northern Thailand indicates that ITS2 sequences of *O. viverrini* exhibit extremely low or no intraspecific variation, with a single haplotype detected across multiple populations, reflecting strong sequence conservation at this locus [35]. This limited variability restricts the utility of ITS2 for population-level analyses, despite its effectiveness for species discrimination.

### 3.2. Microsatellite Markers

Further advances in molecular approaches have been achieved through the use of highly polymorphic microsatellite markers. Microsatellites, or simple sequence repeats (SSRs), are short tandemly repeated DNA motifs (typically 1–6 bp) that are widely distributed throughout the genome. Because they exhibit high mutation rates, microsatellites often show extensive allelic diversity, making them particularly informative for population genetic analyses [37]. These properties make microsatellites especially powerful for detecting fine-scale population structure and estimating gene flow among populations. Microsatellite-based studies of *O. viverrini* in Thailand and Lao PDR have revealed pronounced genetic differentiation even at relatively small spatial scales. In some cases, parasite populations separated by short geographic distances displayed distinct allele frequencies and significant *F*_ST_ values, indicating genetic differentiation, restricted gene flow, and localized transmission dynamics.

One of the key insights from microsatellite analyses of *O. viverrini* is the frequent occurrence of heterozygote deficiency, which has been interpreted as evidence for predominant self-fertilization [18]. This reproductive mode can reduce effective recombination and promote genetic differentiation among populations. In addition, the detection of locality-specific alleles suggests that both genetic drift and local adaptation contribute to population divergence. Subsequent studies have further demonstrated that variation in cyprinid fish species, which serve as second intermediate hosts, may influence parasite genetic diversity.

Additional evidence suggests that host-associated transmission pathways may also contribute to genetic structuring in *O. viverrini*. A distinct cat-associated genotype has been identified in Thailand, with mitochondrial markers indicating close genetic similarity between parasites recovered from domestic cats and metacercariae isolated from non-cyprinid fish hosts [38]. These findings raise the possibility that certain parasite populations may utilize alternative transmission pathways that differ from the classical cyprinid-based life cycle. Such host-associated transmission dynamics may contribute to localized parasite adaptation and the maintenance of cryptic population structure within endemic regions.

Integrative analyses combining mitochondrial, nuclear, and microsatellite markers have also revealed a genetically distinct cryptic population from Phang Khon District in Sakon Nakhon Province, Thailand [18,30]. Together, these findings emphasize the importance of both life-history traits and ecological factors in shaping parasite population structure. Importantly, many of the broad patterns first suggested by allozyme analyses—including geographic subdivision, restricted gene flow, and concordant structuring between parasite and snail hosts—have subsequently been supported and refined by higher-resolution DNA-based markers.

### 3.3. Nuclear Introns: Higher-Resolution Emerging Markers

More recently, nuclear intron markers have emerged as powerful tools for resolving genetic variation in *O. viverrini*. As non-coding regions, introns are subject to fewer selective constraints and can accumulate substantial polymorphism. For example, introns of the taurocyamine kinase (TK) gene, such as TkD1Int5, exhibit high variability, including insertions, deletions, and microsatellite-like repeats [39]. These highly variable markers have also provided new insights into the epidemiological role of animal reservoirs. Analyses of *O. viverrini*-like eggs from domestic animals in northeastern Thailand revealed multiple novel haplotypes, some aligning with known lineages and others representing distinct genetic groups. This suggests that non-human hosts, particularly cats and dogs, contribute to maintaining parasite genetic diversity and may facilitate transmission across ecological boundaries. Collectively, these findings underscore the importance of incorporating multi-host perspectives in population genetic studies of trematodes [40].

### 3.4. Synthesis and Emerging Perspectives

Despite advances in the development of DNA-based molecular markers to investigate phylogeographic structure, several challenges remain. Many studies have relied on single or limited numbers of genetic loci, which may not fully capture the complexity of parasite population dynamics. In addition, variation in sampling design, marker choice, and analytical approaches complicates direct comparisons among studies. To facilitate a clearer comparison of methodological approaches, the key characteristics, strengths, and limitations of commonly used genetic markers in *O. viverrini* research are summarized in Table 1. Furthermore, the integration of genetic data with ecological and epidemiological information remains limited, constraining our ability to translate genetic insights into effective control strategies.

Collectively, DNA-based studies have fundamentally transformed our understanding of *O. viverrini* population structure. Rather than representing a single panmictic species, evidence suggests the parasite is a geographically structured complex of genetically differentiated populations distributed across the Greater Mekong Subregion. Importantly, the consistency of patterns across independent marker systems strengthens the inference that hydrological connectivity, host ecology, and landscape heterogeneity play central roles in shaping parasite population structure rather than simply reflecting methodological artefacts alone.

## 4. Genetic Structure of Bithynia Snail Hosts

Snails of the genus *Bithynia* are widely distributed in freshwater ecosystems throughout much of mainland Southeast Asia and play a central role in sustaining the life cycle of *O. viverrini*. As the first intermediate host, *Bithynia* snails support the asexual development and amplification of the parasite, during which miracidia develop into sporocysts, rediae, and ultimately cercariae. The latter are released into aquatic habitats to infect cyprinid fish. As this multi-stage developmental process is indispensable for parasite transmission, understanding the population structure, genetic diversity, and dispersal patterns of *Bithynia* snails is fundamental for elucidating spatial variation in parasite transmission dynamics and epidemiology [5,18].

Historically, the taxonomy of *Bithynia* snails relied primarily on shell morphology and geographic distribution. Traditional classifications recognized several subspecies within *Bithynia siamensis* sensu lato, including *B. s. siamensis* and *B. s. goniomphalos*, which were distinguished primarily by differences in shell shape, size, subtle morphological characteristics, and geographic distribution, with *B. s. siamensis* occurring predominantly in central Thailand and *B. s. goniomphalos* mainly distributed in northeastern Thailand [42]. However, morphological variation in gastropods can be strongly influenced by environmental factors such as water chemistry, habitat type, and food availability [43]. As a result, shell characteristics alone may not reliably reflect underlying evolutionary relationships. Phenotypic plasticity can therefore obscure true genetic boundaries among populations and complicate taxonomic interpretation [44]. These limitations have motivated the increasing use of molecular approaches to clarify the systematics and population structure of medically important *Bithynia* lineages.

Molecular approaches have increasingly been applied to investigate the genetic diversity and phylogeography of *Bithynia* snails, revealing complex patterns of variation across Southeast Asia. Early evidence for a species complex and possible host–parasite co-divergence was provided by Saijuntha et al. [24]. Using allozyme markers, that study demonstrated concordant genetic structuring between *O. viverrini* and its *Bithynia* snail hosts across distinct wetland systems, suggesting patterns of co-divergence associated with the spatial separation and hydrological connectivity of wetland and river systems. Subsequent work further confirmed substantial cryptic diversity within *Bithynia*, showing high genetic divergence (67–73% fixed differences) among taxa and indicating that some subspecies may represent distinct species. 

**Figure 1:**
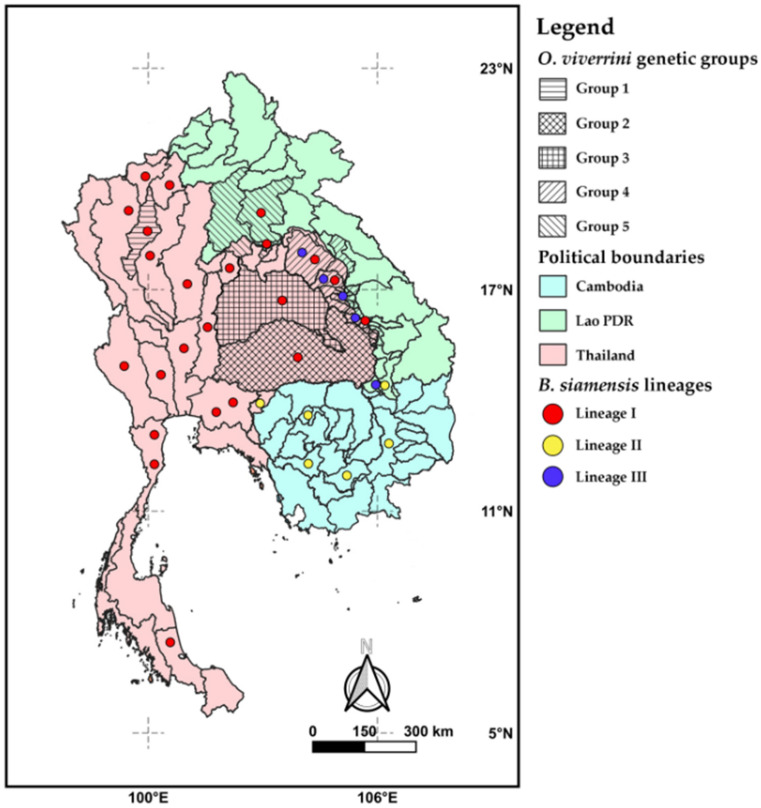
Map showing the geographic distribution of genetic lineages (I–III) of *Bithynia siamensis* sensu lato across Thailand, Lao PDR, and Cambodia based on mitochondrial *CO1* and 16S rDNA sequence analyses. Colored circles indicate the three *B. siamensis* lineages: Lineage I (red), Lineage II (yellow), and Lineage III (blue). Different shaded pattern areas represent the five genetic groups of *Opisthorchis viverrini* identified in previous population genetic studies [24]. Black lines indicate major river and wetland boundaries within the Lower Mekong Basin. Different background colors denote political boundaries: Thailand (pink), Lao PDR (green), and Cambodia (light blue). Spatial data processing, analysis, and map production were carried out using QGIS version 3.34 (QGIS Development Team). The final maps were prepared using the layout tools available in QGIS 3.34 [46].

Subsequently, mitochondrial markers, particularly the *CO1* gene and 16S rDNA region, were widely applied for molecular identification and population genetic analyses of bithyniid snails in Thailand, revealing high haplotype diversity among *Bithynia* populations across Thailand and neighboring countries [45]. These studies have consistently demonstrated strong population subdivision. For example, *B. siamensis* sensu lato is structured into at least three evolutionary lineages (Figure 1) rather than representing a single homogeneous species complex [19,20]. Interestingly, *B. s. siamensis* was closely aligned with lineage I of *B. s. goniomphalos*, suggesting a lack of clear genetic differentiation between these taxa and supporting the hypothesis that they may constitute a single genetically structured species complex rather than distinct subspecies [20].

These genetically distinct lineages correspond to specific river catchments or wetland systems within the region [19]. These patterns suggest that hydrological connectivity strongly influences dispersal and gene flow among *Bithynia* populations, while watershed separation likely contributes to long-term genetic divergence among snail lineages [47,48]. Because *Bithynia* snails are essential for the development and transmission of *O. viverrini*, hydrologically structured variation in snail populations may have important implications for the epidemiology, distribution, and persistence of parasite populations. Consequently, patterns of genetic subdivision in snail hosts may contribute to localized transmission dynamics, whereas connected aquatic systems may facilitate broader parasite dispersal and transmission connectivity across endemic landscapes [49].

Furthermore, understanding the genetic structure of *Bithynia* snails can help clarify the evolutionary relationships within this group and improve the identification of cryptic species [18]. In many freshwater gastropods, molecular analyses have revealed hidden diversity that is not apparent from morphology alone [50,51]. Similar patterns may occur within the *B. siamensis* complex, where distinct genetic lineages may represent previously unrecognized taxa or locally adapted populations. Continued integration of molecular, morphological, and ecological data will therefore be essential for resolving the systematics of these medically important snails and for clarifying their role in sustaining the transmission of *O. viverrini* [18].

## 5. The Transition to Population Genomics

The rapid development of next-generation sequencing (NGS) technologies has ushered in a new era in the study of parasite evolution. Traditional population genetic studies of parasites relied on a limited number of genetic markers, such as mitochondrial genes or a few nuclear loci. While these approaches provided important insights into genetic diversity and geographic structure, they often lacked sufficient resolution to fully reconstruct evolutionary processes or detect fine-scale population differentiation [52]. Population genomic approaches now allow researchers to examine genome-wide patterns of variation across thousands of loci simultaneously, providing far greater power to investigate parasite evolution and transmission dynamics.

Modern genomic methods, including whole-genome sequencing, reduced-representation sequencing techniques (e.g., RAD-seq), and single nucleotide polymorphism (SNP) genotyping, are increasingly being applied to helminth parasites [52,53]. These approaches enable genome-wide analysis of genetic variation, allowing researchers to quantify gene flow among populations, identify loci potentially associated with natural selection, and reconstruct historical demographic processes such as population expansion or fragmentation. Such genome-scale analyses provide unprecedented resolution for investigating how parasite populations evolve and respond to ecological and environmental change [52,54].

The whole genome of *O. viverrini* has recently been characterized at chromosome-level resolution, representing a significant advancement over earlier draft assemblies. This genome comprises approximately 627 Mb, with the majority of sequences scaffolded into six chromosomal pseudomolecules, providing a high-quality reference for downstream genomic and evolutionary studies [41]. This improved genomic resource enables more robust analyses of genome-wide variation, facilitates the identification of functional genes, and supports the application of genomic epidemiology to better understand parasite transmission, population connectivity, and patterns of evolutionary divergence across endemic regions.

In the case of *O. viverrini*, genomic analyses hold great promise for uncovering the molecular basis of host–parasite interactions. Genome-wide studies have identified numerous parasite-derived proteins, including secreted molecules such as granulins, cathepsins, and venom allergen-like proteins, that are implicated in host tissue interaction, immune modulation, and parasite-induced carcinogenesis [55,56].

Comparative genomic and transcriptomic analyses further enable the identification of genes expressed across different life stages and host environments, revealing over 2000 genes associated with infection and host adaptation [57]. Such genome-scale data provide critical insights into how parasite populations adapt to diverse ecological conditions and host systems, ultimately shaping transmission dynamics and persistence in endemic regions. Similarly, genomic approaches applied to *Bithynia* snail hosts could provide new insights into the genetic factors underlying susceptibility or resistance to trematode infection. Identifying snail genes associated with parasite compatibility could help explain why certain snail populations are more effective at supporting transmission than others. Integrating genomic data from both parasite and host populations may therefore provide a more comprehensive understanding of the coevolutionary dynamics shaping this host–parasite system and the ecological processes that structure transmission across endemic landscapes [55].

In contemporary times, human-driven environmental change has been altering the river and wetland systems that structure parasite transmission. Across the Mekong Basin, hydropower development, irrigation expansion, aquaculture intensification, and climate variability have transformed hydrological connectivity between wetland ecosystems [58,59]. Again, because both parasite and snail populations are closely linked to aquatic connectivity, these environmental changes may influence in an evolutionary context, patterns of gene flow and population structure [60]. For example, the construction of dams and roads may isolate populations that were previously connected through river networks, while irrigation systems and aquaculture activities may create new dispersal pathways for hosts and parasites [61]. Consequently, ongoing environmental modification may not only alter contemporary transmission dynamics but may also reshape phylogeographic patterns that emerged through long-term interactions among hydrological connectivity, host dispersal, and evolutionary divergence across endemic landscapes.

Landscape genomics offers a powerful framework for examining how environmental factors interact with genetic variation across complex ecosystems. By integrating genomic data with spatial and environmental information, researchers can investigate how landscape features influence host–parasite evolution and disease transmission. Such integrative approaches will be essential for understanding how ecological change shapes the future dynamics of opisthorchiasis and for developing adaptive strategies to control this important parasitic disease. These approaches also provide a framework for reconstructing phylogeographic structure and examining how long-term hydrological connectivity, watershed isolation, and environmental change shape evolutionary divergence across endemic landscapes.

## 6. Host–Parasite Coevolution

One of the most intriguing findings emerging from recent population genetic studies is the concordance between the population structures of *O. viverrini* and *Bithynia* snails [25]. Genetic patterns observed in parasite populations often show broad concordance with those found in snail populations across endemic regions of the Lower Mekong Basin (Figure 1). Such congruent phylogeographic patterns suggest that parasite and host lineages may have co-diverged within shared aquatic landscapes over evolutionary time. This observation is consistent with the concept of host–parasite coevolution, in which reciprocal selective pressures between interacting species drive adaptive evolutionary change [18]. In such systems, parasite populations may become locally adapted to particular host lineages, while hosts may evolve corresponding defensive mechanisms in response to parasite infection. At the same time, the geographic structuring of both host and parasite populations suggests that long-term drainage organization, ecological isolation, and aquatic connectivity may also have played important roles in shaping opportunities for host–parasite interaction, dispersal, and divergence across endemic landscapes.

If parasite lineages are adapted to specific snail populations, this interaction could reinforce genetic structure across landscapes and contribute to the formation of locally adapted host–parasite assemblages. In practical terms, variation in susceptibility among snail lineages may influence the efficiency of parasite transmission in different geographic areas. For example, certain *Bithynia* populations may be more compatible with particular parasite genotypes, allowing successful development of larval stages within the snail host [21]. Conversely, other snail populations may be less permissive, thereby reducing parasite transmission. Consequently, the distribution of genetic diversity in snail hosts could directly shape parasite population structure and influence local patterns of infection risk. Understanding these coevolutionary dynamics is therefore important for interpreting the spatial distribution of *O. viverrini* transmission across endemic regions. 

Because the life cycle of the liver fluke involves multiple hosts, each host stage potentially imposes distinct ecological and evolutionary pressures on parasite populations. For example, differences in susceptibility among fish species could influence which parasite genotypes successfully reach the metacercarial stage. Similarly, the mobility, feeding behavior, and habitat preferences of definitive hosts may facilitate the dispersal of parasites across different aquatic environments [12,18]. Conversely, isolated wetlands or fragmented habitats may limit host movement and promote genetic differentiation among parasite populations [62]. Together, these ecological and environmental processes likely interact with host-associated factors to shape the complex population structure observed in *O. viverrini* across endemic landscapes. 

Furthermore, numerous aquatic and terrestrial animals participate in different stages of the parasite life cycle, creating a highly complex transmission network. Fish communities vary among habitats, and domestic or wild mammals may contribute differently to parasite dispersal and transmission persistence. Such ecological complexity suggests that the population structure of *O. viverrini* likely reflects the combined influence of host ecology, environmental heterogeneity, and the spatial organization of aquatic habitats [12,18]. Integrating genetic data from parasites with ecological information on snail, fish, and mammalian hosts will therefore be essential for understanding how host–parasite interactions shape transmission dynamics and evolutionary processes in this medically important parasite [5,18,62].

## 7. Implications of Genetic Variation for Understanding Transmission Dynamics

Understanding genetic variation in *O. viverrini* and its intermediate hosts provides important insights into how transmission systems are spatially organized, maintained, and connected across endemic landscapes. Despite decades of control efforts focusing mainly on chemotherapy (praziquantel administration) and health education, opisthorchiasis remains highly endemic in northeastern Thailand and neighboring countries [63,64]. Population genetic studies offer an additional perspective by revealing how parasite populations are structured across landscapes, how they interact with their hosts, and how transmission networks are maintained [18,60].

One key contribution of population genetic research is the identification of spatial structure in parasite populations. Early studies using allozyme markers suggested genetic differentiation among parasite populations in different regions [25]. Later analyses using mitochondrial and nuclear DNA markers confirmed that *O. viverrini* populations are not always panmictic but can show geographic structuring associated with river basins, ecological barriers, or hydrological connectivity [5,18]. Such patterns indicate that parasite dispersal may be constrained by environmental factors, leading to localized transmission cycles. Recognizing these genetic subdivisions can help define epidemiological units and improve understanding of how transmission is maintained and potentially re-established across endemic landscapes.

Genetic variation also has important ecological implications for the relationship between *O. viverrini* and its first intermediate hosts, snails of the genus *Bithynia* [18]. Studies have demonstrated considerable genetic diversity among *Bithynia* snail populations across Southeast Asia [20]. Interestingly, patterns of population structure in parasite populations often correspond with those observed in snail hosts, suggesting possible host–parasite coevolution or local adaptation [25]. If parasite lineages are adapted to specific snail populations, transmission success may depend on the compatibility between parasite genotypes and local host populations. Consequently, the spatial distribution of snail genetic diversity could influence parasite population structure and broader transmission dynamics.

These host–parasite genetic interactions also have important implications for understanding how transmission systems persist across endemic landscapes. Traditional opisthorchiasis control programs primarily focus on reducing human infection through mass drug administration and behavioral change, particularly discouraging the consumption of raw or undercooked fish [65]. While these measures are essential, they do not fully address the ecological processes that maintain parasite transmission in natural environments. Understanding the genetic structure of snail populations and their compatibility with parasite lineages may help identify environments where transmission is more likely to persist. Because the transmission cycle of *O. viverrini* depends on aquatic hosts, hydrological systems such as rivers, wetlands, and irrigation canals may facilitate host and parasite dispersal, whereas isolated water bodies or barriers may restrict movement and promote local genetic differentiation [59]. Integrating population genetics with landscape ecology can therefore improve understanding of how transmission is maintained, dispersed, and reconnected across endemic landscapes [66].

Recent advances in genomic technologies provide new opportunities for studying parasite populations at much higher resolution. Genome-scale analyses can reveal fine-scale population structure, identify genes associated with host compatibility or pathogenicity, and track the movement of parasite lineages across regions. In *O. viverrini*, genomic resources have enabled the identification of genes involved in host–parasite interactions, immune modulation, and parasite-induced carcinogenesis [54,55]. These approaches may also support molecular epidemiology by helping distinguish between persistent local transmission and the reintroduction of parasite lineages from other areas. Such information improves understanding of transmission persistence.

Overall, integrating parasite population genetics with host ecology, landscape features, and human behavior provides a more comprehensive framework for understanding *O. viverrini* transmission across endemic landscapes. As genomic technologies continue to advance, integrative studies examining both parasite and host populations will play an increasingly important role in clarifying how transmission systems persist, interact, and evolve across endemic landscapes.

## 8. Conclusions

Over the past two decades, research on *O. viverrini* and its *Bithynia* hosts has fundamentally reshaped our understanding of opisthorchiasis transmission in Southeast Asia. What was once regarded as a single, broadly distributed, and genetically homogeneous parasite is now recognized as a geographically structured species complex shaped by hydrological organization of river basins, host dispersal, and ecological heterogeneity across the Mekong region. Evidence from allozymes, mitochondrial and nuclear markers, microsatellites, introns, and emerging genomic approaches consistently indicates that parasite populations are organized across interconnected aquatic landscapes rather than occurring as a single panmictic transmission system.

At the same time, concordant genetic structuring between parasite and snail hosts suggests that long-term host associations, dispersal pathways, and hydrological connectivity have played important roles in shaping transmission dynamics and evolutionary divergence. These findings increasingly support a view of opisthorchiasis as a spatially structured ecological system embedded within river basins, wetlands, reservoirs, and floodplain networks. Consequently, transmission persistence, dispersal, and reintroduction may depend as much on landscape connectivity and host movement as on local infection prevalence alone.

## 9. Future Perspectives

Future research should move beyond single-marker and single-host approaches toward integrative frameworks that combine population genomics, hydrology, landscape ecology, and multi-host epidemiology. Such approaches will be essential for identifying transmission corridors, resolving cryptic diversity, understanding host–parasite compatibility, and predicting how environmental change may alter disease dynamics across endemic regions. Ultimately, integrating evolutionary, ecological, and hydrological perspectives may provide a stronger foundation for understanding how transmission systems persist, disperse, reconnect, and respond to environmental change across endemic landscapes in an era of rapid environmental transformation.

## Figures and Tables

**Table 1:** Summary of molecular markers used in population genetic studies of *Opisthorchis viverrini* and *Bithynia* snails, highlighting their strengths, limitations, and key contributions to understanding parasite population structure and phylogeography.

Marker Type	Examples	Strengths	Limitations	Key Findings	
				*O. viverrini*	*Bithynia* Snails
Allozymes	Enzyme loci	First molecular evidence of genetic variation; simple and cost-effective	Low resolution; limited loci; cannot detect silent/non-coding variation	Revealed early regional differentiation; challenged panmictic assumption [24,25]	Provided initial evidence of population structure and geographic differentiation [24]
Mitochondrial DNA	*CO1*, *ND1*, *cyt-b*, 16S rDNA	High mutation rate; sensitive to recent divergence; easy amplification	Maternal inheritance; single locus; affected by introgression and selection	Low variation was observed; host-specific genotypes identified [28,29,30,31]	Revealed high haplotype diversity and clear lineage structuring associated with hydrological systems [19,20,21]
Nuclear rDNA	ITS1, ITS2	Useful for species identification; complements mtDNA	Relatively low variability; limited resolution at population level	Low variation was detected, but it remains suitable for species differentiation [34,35]	N/A
Microsatellites	SSR markers	Highly polymorphic; suitable for fine-scale population structure	Null alleles; homoplasy; scoring complexity	Detected micro-geographic structure; evidence of self-fertilization [18]	N/A
Nuclear introns	TK introns (e.g., TkD1Int5)	High variability; biparental inheritance; informative for population genetics	Alignment complexity; indels complicate analysis	Revealed high haplotype diversity and heterozygosity, indicating genetically distinct groups [39,40]	Revealed high haplotype diversity and heterozygosity [18]
Genomics	SNPs, RAD-seq, WGS	Genome-wide resolution; detects selection, gene flow, demographic history	Higher cost; computational demands; requires bioinformatics expertise	Emerging approaches for resolving fine-scale structure and adaptive variation [41]	N/A

N/A, not analyzed.

## Data Availability

No new data were created or analyzed in this study. Data sharing is not applicable to this article.
